# Enhancing the production of *S*-adenosyl-L-methionine in *Pichia pastoris* GS115 by metabolic engineering

**DOI:** 10.1186/2191-0855-2-57

**Published:** 2012-10-30

**Authors:** Ping Yu, Xiaoqin Shen

**Affiliations:** 1 College of Food Science and Biotechnology, Zhejiang Gongshang University, 149 Jiaogong Road, Hangzhou, 310035, Zhejiang Province, People’s Republic of China

**Keywords:** *S*-adenosyl-L-methionine, *Pichia pastoris* GS115, Metabolic engineering, Fermentation

## Abstract

*S*-adenosyl-L-methionine is an important bioactive molecule participating in a number of biochemical reactions including the transmethylation and transsulphuration reactions of proteins and the biosynthesis of aliphatic polyamines. Strategies of metabolic engineering were used to alter the metabolic flux for enhancing the production of *S*-adenosyl-L-methionine (SAM) in *Pichia pastoris* GS115. These strategies include the over-expression of *Sam*2 by knock-in technique and the disruption of *Cbs* by knock-out technique. Three strains, ZJGSU1 with knock- in of *Sam*2, ZJGSU2 with knock-out of *Cbs* and ZJGSU3 with both knock-in of *Sam*2 and knock -out of *Cbs*, were constructed for the effective production of SAM. Yields of SAM in strains ZJGSU1 and ZJGSU2 were 32- and 5-fold higher than in the original strain *P. pastoris* GS115, respectively. The strain ZJGSU3 had a dramatic increase in the SAM yield, and it was 46-fold higher compared to the original strain. These results indicate that there is a strong synergistic effect on the production of SAM by combining knock-in with knock-out techniques. The yield of SAM in ZJGSU3 strain was 4.37 g/L in a 3 L fermentor. This study provides deep insight into the effective industrial production of SAM in future.

## Introduction

*S*-adenosyl-L-methionine (SAM ) plays a significant role in many biological processes since it is a major methyl group donor in the transmethylation and transsulphuration reactions of proteins, nucleic acids, polysaccharides and fatty acids (Cantoni, 1953; Meister et al. 1984). SAM is very effective in the treatment of osteoarthritis, affective disorders and liver diseases (Barcelo et al. 1990; Lieber, 1999). Recently, SAM as an intracellular bioactive small molecule has deserved more attentions due to its critical roles in human health and has been successfully used in human therapy for the depressive syndrome and the osteoarthritis (Barcelo et al. 1990; Cimino et al. 1984; Osman et al. 1993).

SAM is prepared commercially by the extraction of yeast cells cultured in media supplied with L-methionine (L-Met) (Schlenk et al. 1965; Shiomi et al. 1990). Two isozymes of SAM synthase, SAM1 synthase and SAM2 synthase which are respectively encoded by genes *Sam*1 and *Sam*2, have been identified in *Saccharomyces cerevisiae*. *Sam*1 transcription can be inhibited with excessive L-Met while *Sam*2 not (Thomas et al. 1988). *S. cerevisiae* has been one of the most commonly used strain for the production of SAM so far (Liu et al. 2006; Schlenk et al. 1965; Shen et al. 2008; Shiomi et al. 1990; Wang and Tan, 2008; Wang et al. 1965). However, it is not a best choice for over-expressing SAM synthase and overproducing SAM due to the presence of ethanol during culture, which often makes SAM2 ineffective and hence lowers the yield of SAM. In addition, many strains belonging to *Saccharomyces sake* and *Kluyveromyces lactis* have also been studied for the production of SAM (Mincheva et al. 2002; Shiozaki et al. 1989).

**Figure 1:**
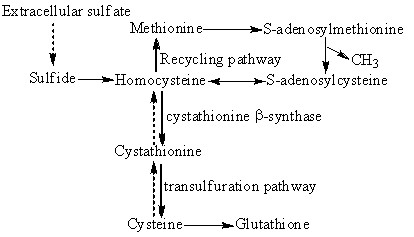
**Metabolic network of homocysteine in *P. pastoris *GS115 strain.** The illustration represents the homocysteine recycling and the transsulfuration pathway. Solid lines indicate reactions that are common in both yeast and mammals, whereas dotted lines are unique to yeast.

In the past twenty years, *Pichia pastoris* has been developed as an excellent host for the high- level expression of the heterologous gene using a strong methanol-controlled alcohol oxidase promoter. It has a potential for the high protein expression level, the efficient secretion of products and the easy culture to a high cell density (Cerehino and Cregg, 2000). The genome sequencing of *P. pastoris* GS115 has been completed and the biosynthetic pathways of many chemicals in it have been elucidated (Schutter et al. 2009). In preference of the chemical or enzyme synthesis, *P. pastoris* is a better candidate to produce the high-level heterologous protein and an industrial strain used widely to produce valuable biochemical molecules (Gross et al. 1983; Matos et al. 1987; Park et al. 1996). In *P. pastoris* GS115, homocysteine has three alternative metabolic fates: to be remethylated to form methionine, or to be combined with serine to form cystathionine via the cystathionine-β-synthase (CBS) reaction, or to revert back to *S*- adenosylhomocysteine (SAH) via reversal of the SAH hydrolase reaction (Figure 1) (Christopher et al. 2002; Wang et al. 2009). From viewpoint of the metabolic flux, it is possible that the over- expression of *Sam* will improve the accumulation of SAM. It has been confirmed by a study conducted by Li et al. ( 2002) in which the introduction of a pPIC3.5k-based *Sam*2 into *P. pastoris* GS115 boosted the production of SAM. Furthermore, since the cystathionine-β-synthase (CBS) acts on the removal of homocysteine from SAM cycle, the disruption of cystathionine-β- synthase gene (*Cbs*) is likely to lead to a high production of SAM in *P. pastoris* GS115. Therefore, it is very interesting to investigate whether both over-expression of *Sam*2 and disruption of *Cbs* in *P. pastoris* GS115 will have a coordinative effect on the production of SAM and result in a higher yield of SAM for the industrial production.

In the present study, three genetically modified *P. pastoris* strains, ZJGSU1 with over- expression of *Sam*2, ZJGSU2 with disruption of *Cbs* and ZJGSU3 with both over-expression of *Sam*2 and disruption of *Cbs*, were constructed and yields of SAM in these three strains in flasks were determined and compared. Over-expression of *Sam*2 and disruption of *Cbs* in *P. pastoris* GS115 show a significantly synergistic effect on the production of SAM. The SAM production in ZJGSU3 strain were also investigated in a 3L fermentor.

## Materials and methods

### Materials

*S. cerevisiae* strain (CCTCC No. M209272) was purchased from the China Center for Type Culture Collection. *P. pastoris* GS115 and the vectors pPIC9K and pPICZα were purchased from the Invitrogen Co. Ltd, USA. PCR reagents, restriction endonucleases and the vector pMD19T were purchased from the TaKaRa Biotech Co. Ltd, Japan. Zeocin, SAM standard sample, G418, histidine and glutathionine were purchased from the Sigma, Co. Ltd. Multicopy *Pichia* Expression Kit was purchase from the Invitrogen Co. Ltd. Yeast nitrogen base and PTM1 trace metals solution were purchased from the Shanghai Sangon Bioengineering Co. Ltd, China. All other reagents were analytical grade and they were used as supplied. Primers used for the amplification of genes and their detection are listed in Table 1.

**Table 1:** Oligonucleotide sequences used as primers

**Names**	**Sequences**
Sam2-F	5’-caggatccaccatgaccaagagcaaaact-3’
Sam2-R	5’-gcggccgcgaattcagcctagcataaagaaa-3’
Zeocin-F	5’-ggactagtagaccttcgtttgtgc-3’
Zeocin-R	5’-ggactagtcggttcctggccttttg-3’
Cbs-F	5’-ttctggagcacattggaa-3’
Cbs-R	5’-agtgtatgcctagatgg-3’
1 F	5’-atcaaagtgctgtagttg-3’
1 R	5’-acacgacctccgaccactcg-3’
2 F	5’-agttagacaacctgaagt-3’
2 R	5’-gataatagttctgtagccct-3’

### Construction of the plasmid pPIC9K-Sam2 and introduction of *Sam2* into *P. pastoris* GS115

**Figure 2:**
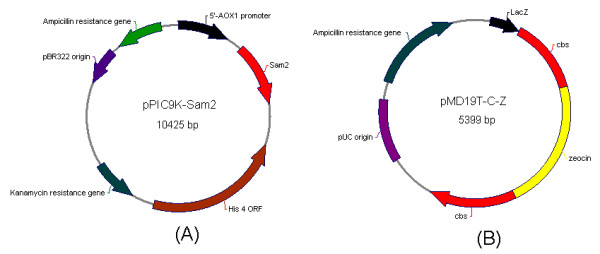
**The maps of recombinant plasmids.** (**A**) The map of the plasmid pPIC9K-Sam2; (**B**) The map of the plasmid pMD19T-C-Z. Sam 2: SAM synthase 2 gene sequence, His 4 ORF: Histidine 4 open reading frame, pBR322 origin: Origin of replication of the plasmid pPIC9K, lac Z: β-galactosidase gene Z, cbs: Cystathionine-β synthase gene, pUC origin: pUC origin of replication, Zeocin: Zeocin resistance gene.

*Sam*2 (GeneBank accession number: gi852113) was amplified using *S. cerevisie* genome as the template and a primer set, Sam2-F and Sam2-R (Table 1). PCR products were purified and digested by *Eco*R I and *Bam*H I, and ligated into the *Eco*RI-*Bam*HI digested plasmid pPIC9K, giving the plasmid pPIC9K-Sam2 (Figure 2A). After linearized by *Bgl* II, the plasmid pPIC9K- Sam2 was transformed into *P. pastoris* GS115 by the electroporation method with parameters: 1.5 kV, 200μF and 200Ω. Transformants were screened by MD plates and G418 plates. The genomic DNA of selected transformants were isolated according to the Multicopy *Pichia* Expression Kit provided by the manufacturer. PCRs were carried out to confirm whether *Sam*2 was integrated into the genomic DNA of *P. pastoris* GS115 strain according to the specification provided by the manufacturer.

### Knock-out of Cbs in *P. pastoris* GS115

*Cbs* (GeneBank accession number: gi853059) was amplified from *P. pastoris* GS115 genome with the primer set, Cbs-F and Cbs-R (Table 2). It was then subcloned into the plasmid pMD19T to form the plasmid pMD19T-CBS. The antibiotic gene of zeocin was amplified from the plasmid pPICZα with the primer set, Zeocin-F and Zeocin-R (Table 2). Amplified zeocin products were digested with *Spe* I and subcloned into the *Spe* I site of the plasmid pMD19T-CBS, giving the plasmid pMD19T-C-Z (Figure 2B). The resultant pMD19T-C-Z was digested with *Bgl* II and *Sal* I, and the 2100 bp fragment C-Z was transformed separately into *P. pastoris* GS115 and ZJGSU1. MD plates supplemented with histidine, zeocin and glutathionine were used to screen transformants. Zeocin positive transformants were confirmed by PCRs with primer sets: 1F and 1R, 2F and 2R, 1F and 2R (Table 2). Transformants with only disruption of *Cbs* and with both introduction of *Sam*2 and disruption of *Cbs* were named ZJGSU2 and ZJGSU3, respectively.

**Table 2:** The activities of SAM synthase in four strains

**Strains**	**Enzyme activity (U/mL)**
GS115	39
ZJGSU1	545
ZJGSU2	309
ZJGSU3	705

### *P. pastoris* culture for the SAM production in flasks

Four strains, *P. pastoris* GS115, ZJGSU1, ZJGSU2 and ZJGSU3, were respectively inoculated into a 5 mL YPD medium and incubated at 30°C for 16 h as seed cultures. A 1mL aliquot of the seed culture was added to 100 mL BMGY medium (yeast extract 1%, peptone 2%, glycerol 1%, 10×YNB10%, 0.1M potassium phosphate buffer, pH 6.0) supplemented with 1% L-Met in 500 mL flasks and cultured at 30°C for 4 d. 1.5% (v/v) of methanol as an inducer and carbon source was added daily for three times after one day of growth. After 96 h of culture, 1 mL of sample was taken out for determining the yield of SAM.

### Fermentation of the ZJGSU3 strain in a 3 L fermentor

The ZJGSU3 strain was grown in a BMGY medium at 30°C for 24 h, 100 mL of cultures were inoculated in a 3 L fermentor containing 1.8 L of minimal salts (PTM) fermentation medium: H_3_PO_4_ (85% stock), 27 mL/L; CaSO_4_, 0.93 g/L; K_2_SO_4_, 18.2 g/L; MgSO_4_·7H_2_O, 14.9 g/L; KOH, 4.13 g/L; Glycerol, 40 g/L and 4.4 mL/L of PTM1 trace metals solution (*P. pastoris* fermentation manual of the Invitrogen). Initial fermentation conditions were as follows: dissolved oxygen (DO) was maintained above 20% by the automatic control of agitation, pH was 5 (adjusted with ammonium hydroxide), temperature was set at 30°C. Cells were grown until glycerol depleted completely. This was indicated by a dramatic increase in the DO to 100%. Glycerol feeding was then initiated to increase the cell biomass under limited conditions: 500 mL of 50% glycerol containing 6 mL of PTM trace salts was fed at 60 mL/h. Following the depletion of glycerol, methanol feeding was initiated at a rate of 10 mL/h and increased gradually to a final rate of 21 mL/h. Meanwhile, 500 mL of the saturated L-Met was fed at a rate of 6 mL/h. Samples were taken out at different times for determining the yield of SAM and biomass.

### Determination of the yield of SAM

The yield of SAM was determined as described by Lin et al. ( 2004). 1 mL of the sample was centrifuged at 12,000 rpm for 10 min, and washed twice with the deionized water, then mixed with 1 mL of 1.5 M HClO_4_ at 4°C for 1.5 h. The supernatant was collected after centrifugation at 12,000 rpm for 5 min and then went through a 0.22 μm filtration membrane before analysis. A 15 μL of the extracted SAM sample was injected into a high performance liquid chromatography (HPLC) system (Agilent, USA) using a C18 column (Hypersil BDS column, 4.6 mm×250 mm, 5 μm) with a mobile phase composed of 0.01 M ammonium formate (pH3.0) at a flow rate of 0.8 mL/min. Peak area analysis was performed based on the standard calibration curve of SAM. Due to the instability of SAM, its *p*-toluenesulfonate salt (Sigma) was used as standard sample.

### Determination of wet cell weight

10 mL of the sample was taken out and centrifuged at 12,000 rpm for 10 min, and then washed three times with the distilled water. The wet cell weight was determined using a electronic balance.

### Determination of the activity of SAM synthase

The activity of SAM synthase was determined as described by Li et al. ( 2002). Cells in 1mL of the sample were harvested by centrifugation at 6000 rpm for 5 min, and washed immediately with ice-cold lytic buffer (50 mM potassium phosphate at pH 7.4, 5% (v/v) glycerol, 5 mM mercaptoethanol, 1 mM EDTA), and then centrifuged at 12,000 rpm for 5 min. Cell pellets were resuspended in 1 mL of the lytic buffer and disrupted by the ultrasonic treatment. The supernatant was collected by centrifugation at 12,000 rpm for 10 min and used as the sample to assay the activity of SAM synthase as follows:1 mL of the reaction mixture, containing 20 mM L-Met, 20 mM ATP, 8 mM reduced glutathione, 20 mM MgCl_2_, 100 mM KCl, 150 mM Tris -HCl and the supernatant with an appropriate concentration, was incubated at 37°C for 1 h. 0.5 mL of 20% HClO_4_ was then added to the reaction mixture. The resultant precipitation was removed by centrifugation at 12,000 rpm for 10 min. The supernatant was used for determining the yield of SAM by the HPLC. One unit of the activity of SAM synthase was defined as the amount of enzyme required to catalyze the transformation of 1μmol of L-Met into SAM per minute at 37°C.

## Results

### Identification of the introduction of *Sam2* or the disruption of *Cbs* and their respective effect on the production of SAM

Over 5000 transformants grew on MD plates after the electro transformation of pPIC9K-sam2 into *P. pastoris* GS115. Transformants having a better growth rate on MD plates were streaked on YPD plates containing different concentrations of G418 (0.5, 1.0, 2.0, 3.0, 4.0 mg/mL). 25 transformants having a faster growth rate were picked from YPD plates with G418 at a final concentration of 4 mg/mL. These transformants were cultured in flasks for evaluating the yield of SAM by HPLC. The transformant which produced the highest yield of SAM was chosen finally and named ZJGSU1. According to the manual of the “multi-copy of *P. pastoris* expression kit” from the Invitrogen, multiple integrated copies can lead to the increase in the G418 resistance level from 0.5 mg/mL (1–2 copies) to 4 mg/mL (7–12 copies). Thus, the copy number of the *Sam*2 gene in ZJGSU1 strain was estimated to be 7–12 based on its resistance to G418 at a final concentration of 4 mg/mL. The yield of SAM in ZJGSU1 strain reached 0.8 g/L.

**Figure 3:**
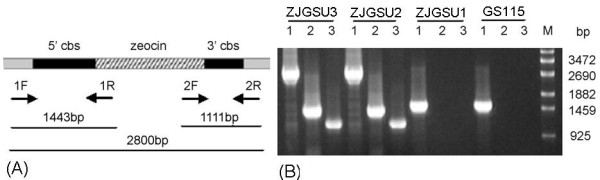
**(A) The map of the C-Z gene fragment. Primers 1F, 2F, 1R and 2R were designed based on this fragment.** If the homologous recombination happens, PCR product using the primer set, 1F and 1R, is a 1443-bp fragment between 5’cbs and zeocin (PCR1). PCR product using the primer set, 2F and 2R, is a 1111-bp fragment between 3’cbs and zeocin (PCR2). PCR product using the primer set, 1F and 2R, is a 2800-bp fragment including cbs and zeocin (PCR3). (**B**) Identification of the *Cbs* disruption with PCRs. The genomic DNA was respectively extracted from different strains. The crossover of 5’ was identified by PCR1, the 3’crossover was identified by PCR2, and the knock-out of *Cbs* by the homologous recombination was identified by PCR3.

**Figure 4:**
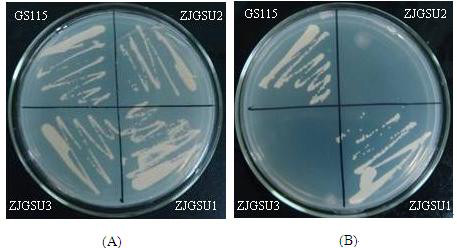
**Identification of four strains on selective MD plates.** (**A**) Strains grow on the MD plate supplemented with both histidine and glutathionine. (**B**) Strains grow on MD plates supplemented with histidine only.

The plasmid pMD19T-C-Z was designed and constructed to disrupt *Cbs* in *P. pastoris* GS115. A knock-out vector (Figure 3A) was also designed to evaluate the effect of the *Cbs* knock-out on the production of SAM. It contains a full length of *P. pastoris Cbs* gene (1500 bp) split by the zeocin expression cassette (1200 bp) in the middle. After the plasmid pMD19T-C-Z was transformed into the host strain, the homologous recombination would occur between the genomic DNA and the transformed DNA. Recombinants could be selected by the antibiotic zeocin. Since zeocin resistance strains might have a single crossover or a random insertion of the transformed DNA, the selected transformant was confirmed by PCR using primer sets, 1F and 1R, 2F and 2R, 1F and 2R (Figure 3B). PCR results demonstrated that the transformed DNA was inserted into the genomic DNA by the homologous recombination. Furthermore, since the dysfunction of *Cbs* in *P. pastoris* GS115 could lead to the cysteine auxotrophy, the growth of these strains was dependent on the addition of cysteine or glutathionine to the medium. The growth of four strains, GS115, ZJGSU1, ZJGSU2 and ZJGSU3, was observed on the medium. As shown in Figure 2, four strains could grow well on the medium with glutathionine (Figure 4A), whereas strains ZJGSU2 and ZJGSU3 which have a *Cbs* knock-out did not on the medium without glutathionine (Figure 4B). This indicates that *Cbs* is disrupted successfully in strains ZJGSU2 and ZJGSU3. The disruption of *Cbs* also contributed to the accumulation of SAM, though not so much as the introduction of SAM synthase. The yield of SAM in the ZJGSU2 strain reached 0.13 g/L.

### Effect of both knock-in of *Sam2* and knock-out of *Cbs* on the production of SAM

**Figure 5:**
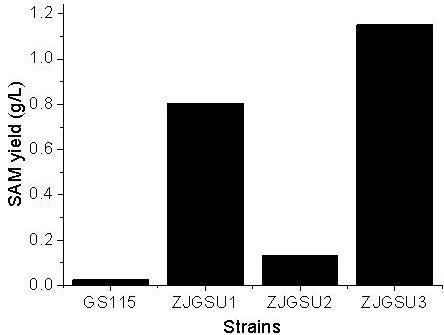
**The yields of SAM in different strains.** Data are represented as the mean of three measures. GS115: *P. pastoris* GS115, ZJGSU1: *P. pastoris* GS115 with knock- in of *Sam*2, ZJGSU2: *P. pastoris* GS115 with knock-out of *Cbs*, and ZJGSU3: *P. pastoris* GS115 with both knock-in of *Sam*2 and knock-out of *Cbs*.

Based on the above results, it is obvious that the over-expression of *Sam*2 or the disruption of *Cbs* has a positive effect on the production of SAM. In order to further investigate the effect of both knock-in of *Sam*2 and knock-out of *Cbs* on the production of SAM, the C-Z fragment from the plasmid pPMD19T-C-Z was transformed into the ZJGSU1 strain to produce the ZJGSU3 strain. The *Cbs* knock-out in ZJGSU3 strain was demonstrated by PCR analysis (Figure 3B). The yields of SAM in four strains, GS115, ZJGSU1, ZJGSU2 and ZJGSU3, are shown in Figure 5. The average yield of SAM in GS115 was 0.025 g/L, and it was 0.13 g/L in ZJGSU2. Thus, the yield of SAM in ZJGSU2 is 5-fold higher than that in the original strain GS115. The yield of SAM in ZJGSU1 was 0.8 g/L when *Sam*2 was introduced, 32-fold higher than in the original strain GS115. The yield of SAM in ZJGSU3 strain reached 1.2 g/L, and was over 1.4-fold higher than that in ZJGSU2 and 46-fold higher than in the original strain GS115. These results indicate that both knock-in of *Sam*2 and knock-out of *Cbs* have a synergistic effect on the enhancement of the yield of SAM.

### Analysis of the activity of SAM synthase

The activities of SAM synthase in strains GS115, ZJGSU1, ZJGSU2 and ZJGSU3 were determined. The result is showed in Table 2. The activity of SAM synthase in ZJGSU3 was up to 705 U/ mL, and was nearly twice than that in ZJGSU2 strain and 18-fold higher than that in the original strain GS115. The activity of SAM synthase in ZJGSU1 strain was 544 U/mL, 14-fold higher than that in the original strain GS115. These results further confirm that enhancing the activity of SAM synthase facilitates the production of SAM. In addition, by comparison of the activities of SAM synthase from four strains, it was also found that the knock-out of *Cbs* could increase the expression of *Sam*2, and hence enhanced the activity of SAM synthase and increased the yield of SAM.

### Fermentation of SAM in a 3L fermentor

**Figure 6:**
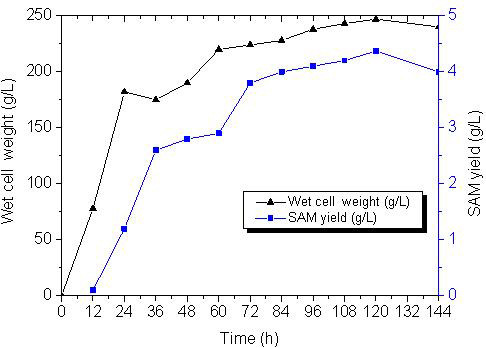
**The growth and production curves of ZJGSU3 strain in a 3L fermentor.** ▲: wet cell weight, ■: the yield of SAM. The X-axis represents the time after the induction with methanol.

Due to a favorable nutrient balance, dissolved oxygen, pH and high cell density in the fermentor, the production of SAM in the ZJGSU3 strain increased greatly as well as the cell growth. The host strain *P. pastoris* GS115 has an unique ability to grow on minimal media to a very high cell density with a strong tightly regulated alcohol oxidase promoter. It allows to accumulate a high level of SAM synthase, and so achieves a high yield of SAM. The glycerol was used as the only carbon source during the first two stages to stimulate the cell growth, which could further enhance the yield of SAM. At the end of the glycerol feeding stage, the wet cell weight was 182 g/L. After the depletion of glycerol, the methanol was then fed to further induce the expression of SAM synthase. By changing the methanol feeding rate, its concentration in the fermentor was maintained at a low level to minimize the toxic effect on the yeast cells. As shown in Figure 6, the maximal biomass and the highest yield of SAM were 247 g/L and 4.37 g/L after 96 h of the methanol induction, respectively.

## Discussion

The results of the present study demonstrate that both introduction of *Sam*2 and disruption of *Cbs* in *P. pastoris* GS115 by knock-in and knock-out techniques have a obviously synergistic effect on the production of SAM, resulting in a significant increase in the yield of SAM. The yields of SAM in ZJGSU3 strain with both introduction of *Sam*2 and disruption of *Cbs* were 1.2 g/L in flasks and 4.37 g/L in a 3 L fermentor, implying that it has a commercial prospect for the large scale industrial production of SAM in future. To our knowledge, this is the first report with regard to the production of SAM by both introduction of *Sam*2 and disruption of *Cbs* in *P. pastoris* GS115 strain using the plasmid pPIC9K as the vector.

Previously, the reports pertinent to the production of SAM focused on the *S. cerevisiae* strain (Liu et al. 2006; Schlenk et al. 1965; Shen et al. 2008; Shiomi et al. 1990; Wang and Tan, 2008; Wang et al. 1965), *S. sake* (Shiozaki et al. 1989) and *K. lacti* (Mincheva et al. 2002). The traditional fermentation is a main way for the production of SAM in those strains. With the development of the genetic and metabolic engineering, enhancing yields of valuable bioactive chemicals by the modern biotechnology becomes an important trend. The yield of SAM was enhanced by altering its metabolic flux in *P. pastoris* GS115 strain in our study. *S. cerevisiae* contains two SAM synthase genes, *Sam*1 and *Sam*2. *Sam*1 is repressed by the excessive L-Met, whereas *Sam*2 is not. Because L-Met is an important precursor for the effective accumulation of SAM, *Sam*2 is chosen for being introduced into *P. pastoris* GS115 strain for enhancing the yield of SAM in this study. In the previous studies, Le et al*.* (2002) and Yu et al*.* ( 2003) reported that the knock-in of SAM synthase gene resulted in the increase in the yield of SAM in *Pichia*. Li et al*.* ( 2002) also reported a constructed strain with knock-out of *Cbs*, but did not investigate the knock-out effect on the production of SAM. Chan and Appling ( 2003) reported that the deletion of *Cbs* in *S. cerevisiae* did not cause a high accumulation of SAM, and the effect of the production of SAM in wild type and mutant strains was not also compared. In our study, the knock-out of *Cbs* in *P. pastoris* GS115 strain not only increases the yield of SAM, but also enhances the expression of SAM synthase, and so alters the metabolic flux of SAM and facilitates its further accumulation. Furthermore, a synergistic effect of both introduction of *Sam*2 and disruption of *Cbs* in the *P. pastoris* GS115 strain by knock-in and knock-out techniques on the production of SAM is for the first time reported in our study. Compared to *S. cerevisiae*, *P. pastoris* is a better candidate for the industrial production of recombinant proteins and valuable biochemical molecules. It can grow easily to a high cell density in a minimal salts medium with methanol as a sole carbon source and ammonium sulfate as a sole nitrogen source. The genetically modified *P. pastoris* (ZJGSU3) reaches a wet cell weight of 247 g/L and produces 4.37 g/L of SAM in a 3 L fermentor.

In conclusion, a genetically modified strain ZJGSU3 for the effective production of SAM was obtained by both knock-in of *Sam*2 and knock-out of *Cbs* in the *P. pastoris* GS115 strain. This strain shows a great potential for the industrial production of SAM. Continuous efforts should be given to further optimize cultural conditions for the large-scale production of SAM in the fermentor and purify it for the application in the fields of biomedicines, chemical engineering and pharmaceuticals.

## Competing interests

The authors declare that they have no competing interests.
